# ERCC1和PKCalpha在非小细胞肺癌中的表达及其临床意义

**DOI:** 10.3779/j.issn.1009-3419.2010.03.13

**Published:** 2010-03-20

**Authors:** 朗 何, 梅 侯, 光明 李, 世民 文, 红 杨, 萍 陈, 聂 徐

**Affiliations:** 1 637000 南充，川北医学院第二临床医学院（四川省南充市中心医院）肿瘤中心 Cancer Center, the Second Clinical Medical School Affiliated to North Sichuan Medical College (Nanchong Central Hospital, Sichuan), Nanchong 637000, China; 2 610041 成都，四川大学华西医院肿瘤中心 Department of Medicine, Cancer Center, West China Hospital, Sichuan University, Chengdu 610041, China; 3 610041 成都，四川省肿瘤医院病理科 Department of Pathology, Sichuan Provincial Cancer Hospital, Chengdu 637000, China

**Keywords:** 肺肿瘤, 预后, ERCC1, 蛋白激酶C-α, 免疫组织化学, Lung neoplasms, Prognosis, ERCC1 protein, human, Protein kinase C-alpha, Immunohistochemistry

## Abstract

**背景与目的:**

剪切修复偶联因子1（Excision-Repair Cross-Complementing 1, ERCC1）是核苷酸外切修复家族中的重要成员，它在核酸损伤修复过程和凋亡过程中起着重要作用。蛋白激酶C-α（Protein kinase Calpha, PKCα）是蛋白激酶C（PKC）的一种同工酶，PKCα调控细胞的转化和增殖，是肿瘤细胞中重要的信号途径。本研究初步探索ERCC1和PKCα在非小细胞肺癌（non-small cell lung cancer, NSCLC）中表达所代表的临床意义。

**方法:**

运用免疫组化方法检测51例NSCLC组织、21例癌旁组织中ERCC1和PKCα的表达，并采用SPSS 13.0软件进行相关统计分析。

**结果:**

ERCC1和PKCα在肿瘤组阳性率明显高于癌旁组（*P* < 0.05）；ERCC1与临床分期和N分期等因素有关，临床Ⅲ+Ⅳ期及N1-2期患者ERCC1阳性率要分别高于Ⅰ+Ⅱ期和N0期患者（*P*=0.011, *P*=0.015）；ERCC1阴性组的5年生存时间高于阳性组（*P* < 0.05）；*Spearman*相关分析提示ERCC1与PKCα之间存在正相关（*r*=0.425, *P*=0.002）。

**结论:**

ERCC1和PKCα可能与NSCLC的发生相关，ERCC1可能与肿瘤的预后相关。ERCC1和PKCα之间可能存在共同作用通路。

## Abstract (en)

**Background and objective:**

Excision repair cross-complementing 1 (Excision-Repair Cross-Complementing 1, ERCC1), an important member of the DNA repair gene family, plays a key role in nucleotide excision repair and apoptosis of tumor cells. Protein kinase C-α (Protein kinase C, PKCα), an isozyme in protein kinase C family, is an important signaling molecule in signal transduction pathways of tumors, which has been implicated in malignant transformation and proliferation. The aim of this study was to explore the clinical significance of ERCC1 and PKCα in non-small cell lung cancer (NSCLC).

**Methods:**

The expression of ERCC1 and PKCα were examined by immunohistochemistry (IHC) in the specimens of 51 cases of NSCLC patients tissue and 21 cases of paracancerous tissue. The relationship between detected data and patients′ clinical parameters was analyzed by SPSS 13.0 software.

**Results:**

The positive expression rate of ERCC1 and PKCα in NSCLC tissues was significantly higher than paracancerous tissues (*P* < 0.05). Expression of ERCC1 was closely related to clinical stage and N stage. The positive rate of ERCC1 was higher in Ⅲ+Ⅳ or N1+N2 stage patients compared with Ⅰ+Ⅱ or N0 stage (*P*=0.011, *P*=0.015). We also found that 5-year survival of negative group of ERCC1 was remarkably higher than that of positive group by *χ*^2^ test (*P* < 0.05). Expression of ERCC1 was positively correlative to PKCα by *Spearman′s* correlation analysis (*r*=0.425, *P*=0.002) in NSCLC.

**Conclusion:**

The results suggest ERCC1 and PKCα might be correlated with the development of NSCLC. ERCC1 might be related to prognosis of NSCLC. There might be existed a mechanism of coordination or regulation between ERCC1 and PKCα.

非小细胞肺癌（non-small cell lung cancer, NSCLC）约占肺癌的80%，其治疗和预后至今仍无突破性进展，故诊断和治疗NSCLC一直是人们关注的热点。剪切修复偶联因子1（Excision-Repair Cross-Complementing 1, ERCC1）是核苷酸外切修复家族中的重要成员，也是核苷酸切除修复（Nucleotide excision repair, NER）途径的关键基因，它在核酸损伤修复过程和凋亡过程中起着重要作用。蛋白激酶C-α（Protein kinase Calpha, PKCα）是蛋白激酶C（Protein kinase C, PKC）的一种同工酶，是蛋白激酶超家族中的成员，多数报道认为PKCα所介导的信号途径是肿瘤细胞中一种重要的信号途径。本研究采用immunohistochemistry（IHC）分析ERCC1和PKCα在NSCLC组织、癌旁组织中的表达，以初步探讨两者的临床意义。

## 1 材料与方法

### 1.1 临床标本

标本来自于四川大学华西医院住院手术的51例NSCLC患者的术后肿瘤标本和相应的21例癌旁组织标本（癌旁肺组织取材距肿瘤边缘5 cm以上）（2001年1月1日-2002年5月31日），术前均未行抗肿瘤治疗。其病例资料通过医院病案科和随访获取，随访率100%。随访以病理确诊之日起逐月记录，随访5年。

### 1.2 主要抗体及试剂

鼠抗人ERCC1单克隆抗体（ZMED公司，产品编号ZM-0138）；鼠抗人PKCα单克隆抗体（北京中杉金桥生物技术公司，产品编号Sc-8393）；Sp-9000通用SP Kit免疫组化染色试剂盒（北京中杉金桥生物技术公司）；第二代即用型免疫组化Elivision^TM^ plus广谱试剂盒（福建迈新生物技术公司）。

### 1.3 实验方法

本实验采用IHC进行ERCC1及PKCα的检测，预实验得出ERCC1最佳IHC方法为两步法（Elivision，过夜），枸橼酸高压锅修复，工作浓度为1:50；PKCα为LsAB法，EDTA水浴修复，工作浓度为1:50。ERCC1的阳性对照采用强阳性表达组织扁桃体，着色为细胞核；PKCα采用结肠癌组织作为阳性对照，着色为细胞浆。

### 1.4 结果判定

ERCC1呈细胞核棕黄色颗粒沉着（图 1），参照Wachters^[1]^的标准：高倍视野（×400）下随机计数300个肿瘤细胞，肿瘤细胞核不染色或染色阳性细胞数 < 10.0%为阴性（-）； > 10.0%判定为阳性（+）。PKCα表现为胞浆内棕黄色颗粒积聚（图 2），参照许良中^[2]^的标准：染色强度分为无色（0），淡黄（1），棕黄（2），棕褐（3）；染色百分比分为阴性（0），阳性细胞≤10.0%（1），11.0%-50.0%（2），51.0%-75.0%（3）， > 75.0%（4）；两者乘积值为：0-3（-），4-5（+），6-7（++）， > 8（+++）。

**1:**
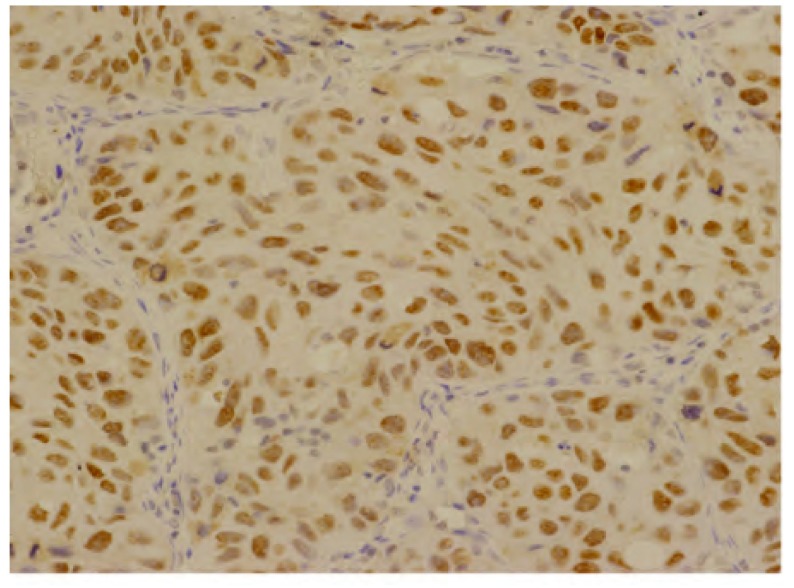
ERCC1在中分化肺鳞癌胞核中的阳性表达（Elivision, ×400） The positive expression of ERCC1 in nucleus of moderately differentiated squamous cell carcinoma of the lung (Elivision, ×400)

**2:**
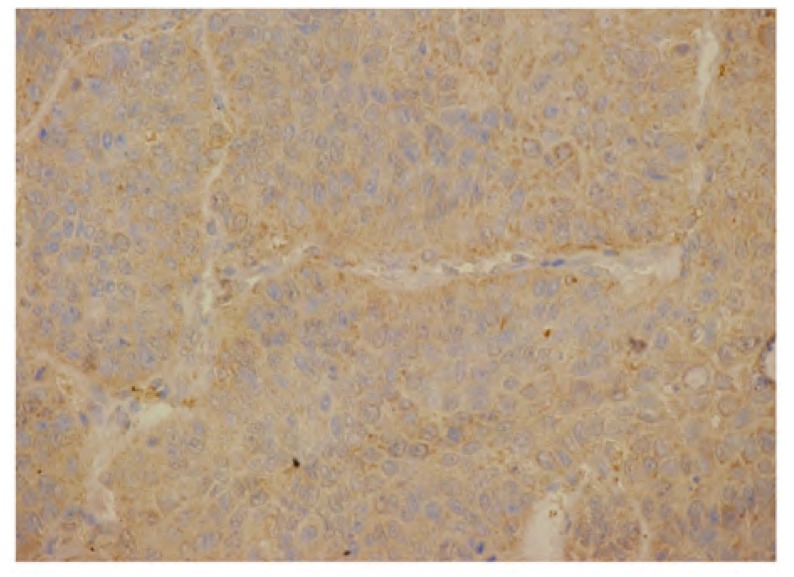
PKCα在中分化肺鳞癌胞浆中的阳性表达（LsAB, ×400） The positive expression of PKCα in cytoplasm of moderately differentiated squamous cell carcinoma of the lung (LsAB, ×400)

### 1.5 统计分析

采用SPSS 13.0统计分析软件，率的比较采用*χ*^2^检验，*Spearman*等级相关分析两因子间关系。*P* < 0.05为差异有统计学意义。

## 2 结果

### 2.1 临床参数

51例NSCLC患者年龄为37岁-82岁，中位年龄为60岁。分别参照2003年WHO肺癌的组织学分类标准和1997年肺癌的UICC分期标准，其中Ⅲ期18例（Ⅲb期患者4例，术前分期为Ⅲa期，术后为Ⅲb期），Ⅳ期5例（包括T3N2M1患者2例与T2N2M1患者1例，为局限性脑转移；余2例患者术前诊断为Ⅲa期，术中发现另一肺叶内存在单个转移性肿瘤结节，术后病理证实为Ⅳ期）（表 1）。

**1:** 51例NSCLC患者临床病理特征 The clinical parameter in 51 patients of NSCLC

Characteristics	*n*
Sex	
Male	40
Female	11
Age	
< 60 years	24
≥60 years	27
Smoking	
Yes	33
No	18
Histology	
Squamous cell carcinoma	21
Adenocarcinoma	24
Adenosquamous carcinoma	6
Differentiation	
Well+Moderate	25
Poor	26
Clinical stage	
Ⅰ+Ⅱ	28
Ⅲ+Ⅳ	23
T stage	
T1+T2	32
T3+T4	19
N stage	
N0	26
N1+2	25
Gross appearance	
Peripheral type	37
Central type	14

### 2.2 ERCC1、PKCα在NSCLC中的表达

NSCLC中ERCC1阳性率为58.8%（30/51），癌旁组织中阳性率为23.8%（5/21），两组阳性率间存在统计学差异（*P*=0.007）。NSCLC中PKCα阳性率为74.5%（38/51），癌旁组织阳性率为42.9%（9 / 21），两组间存在统计学差异（*P*=0.010）。ERCC1表达率证实与不同的临床分期、N分期相关，与性别、年龄、有无吸烟、组织学分型、分化程度、T分期和肿瘤部位等临床病理特征无关，临床Ⅲ+Ⅳ期及N1-2期患者ERCC1表达率要分别高于Ⅰ+Ⅱ期和N0期患者（*P*=0.011和0.015）。PKCα证实与临床各参数间均无关系（*P* > 0.05）。

### 2.3 ERCC1、PKCα染色状态与术后5年生存时间关系

5年随访，生存期 < 5年患者为28例，ERCC1阳性率为71.4%（20/28）；≥5年患者为23例，ERCC1阳性率为43.5%（10/23），*χ*^2^检验证实ERCC1阴性组5年生存时间高于阳性组（*P* < 0.05）；而PKCα阳性组的5年生存时间与阴性组比较并没有差别（*P* > 0.05）（表 2）。

**2:** ERCC1、PKCα阳性组和阴性组5年生存时间的比较 The relationship about 5 years survival between positive group and negative group of ERCC1/ PKCα in NSCLC

Items	ERCC1			PKCα	
	Survival < 5 years (*n*)	Survival≥5 years (*n*)		Survival < 5 years (*n*)	Survival≥5 years (*n*)
Positive	20	10		22	16
Negative	8	13		6	7
Positive rate (%)	71.4	43.5		78.6	69.6
*χ*_ERCC1_^2^=4.073, *P*_ERCC1_ < 0.05; *χ*_PKCα_^2^=0.539, *P*_PKCα_=0.463.					

### 2.4 ERCC1及PKCα之间的关系

*Spearman*相关分析（秩相关分析）发现ERCC1与PKCα之间存在正相关（*r*=0.425, *P*=0.002）。

## 3 讨论

ERCC1是核苷酸外切修复家族中的重要成员，在核酸损伤修复过程中起着重要作用，人类的ERCC1基因具有5′-3′核酸内切酶的活性，故ERCC1缺乏的细胞不能进行铂类-DNA加合物的修复。目前ERCC1与肿瘤的关系正成为研究的热点，已有研究^[3]^证实它可能与肿瘤的发生密切相关。我们通过IHC发现NSCLC标本中ERCC1阳性率为58.8%，此结果与Takenaka^[4]^关于NSCLC、Kwon^[5]^关于晚期胃癌的IHC结果类似，我们的发现支持ERCC1在肿瘤组织中高表达，提示ERCC1可能与NSCLC的发生发展相关。

针对ERCC1与临床参数的关系，本研究发现ERCC1在临床Ⅲ+Ⅳ期及N1-2期患者中高表达，说明ERCC1的阳性状态可能同肿瘤的侵袭性和淋巴结转移关联。而Takenaka^[4]^证实ERCC1阳性表达与肺鳞癌关联；Olaussen^[6]^发现ERCC1表达与患者的年龄、性别、病理类型、胸膜受侵和脉管受侵等明确相关。从上述结果可以看出不同的试验得出了不同的结果，这些差异可能与试验不同的样本量、样本来源以及不同的试验方法有关，故ERCC1与临床参数间的确切关系尚有待大规模的临床试验以明确。对于ERCC1与预后的关系报道各异，已证实ERCC1 mRNA的高表达预示卵巢癌患者疾病进展的高度风险^[7]^。而我们的研究结果与多数结论^[8, 9]^类似，支持ERCC1阴性状态可能与较好的预后相关。但也有不同的结果，Zheng^[10]^即认为ERCC1高表达者的生存时间较长，故ERCC1与NSCLC之间的关系仍需进一步研究。

PKC是细胞内重要的信号转导分子。PKCα属于经典型PKC^[11]^，是PKC的一种同工酶。近来的研究^[12]^结果表明，肿瘤组织中PKCα活性明显增加^[12]^，其异常表达和活化能促进肿瘤细胞的增殖，抑制细胞凋亡，抑制肿瘤细胞的分化。本实验发现NSCLC中PKCα阳性率为74.5%，在肿瘤组织中高表达，与Lahu^[13]^和高志强^[14]^的研究结果类似，这提示PKCα可能参与了肿瘤的发生发展。Byers^[15]^发现特异性siRNA靶向敲除*PKCα*基因能够有效抑制黑素瘤细胞的迁移，说明PKC的效能和表达水平是肿瘤进展的关键因素，但我们的结果却显示出PKCα的染色状态对生存并没有影响（*P* > 0.05），我们认为尚没有依据支持PKCα的表达与NSCLC的生存预后相关。

ERCC1能够识别、切除和修复损伤DNA片段，缺乏ERCC1的细胞会产生DNA修复缺陷，诱发细胞的凋亡反应；PKCα与正常细胞的新陈代谢密切相关，与肿瘤细胞的增殖和细胞凋亡的抑制也有关。到目前为止，国内外尚无这两种基础作用机理不同的检测指标联合运用的报道，考虑到这两种指标可能具有的一些联系，我们初步探索了两者之间的关系，我们发现ERCC1与PKCα之间存在正相关（*P*=0.002），分析可能在肿瘤细胞DNA损伤修复过程与PKC信号传导途径间存在共同通路，从而产生协同或相加效应，对该相关性的进一步深入研究，可能会给NSCLC的治疗提供一条新的途径和一种新的思维。我们的研究表明ERCC1与NSCLC发生和预后相关，且ERCC1与PKCα正相关，但却发现PKCα只与NSCLC的发生相关而与其预后无关，这一看似矛盾的结果可能与不同的实验方法及我们较小的样本量有关，进一步大样本前瞻性研究可能会更清晰明确两者在NSCLC中所扮演的角色及相互关系，因此还需要不断的探索和验证。
